# Supplementary material for: A Likelihood Approach for Real-Time Calibration of Stochastic Compartmental Epidemic Models
Source: PLoS Comput Biol. 2017 Jan 17;13(1):e1005257. doi: 10.1371/journal.pcbi.1005257 (PMC5240920; doi:10.1371/journal.pcbi.1005257)
Supplement: S1 File — (TAR.GZ) [file pcbi.1005257.s014.tar.gz › HSPH_Online-SI-Revision/EnKF07/n1-extreme/n1-extreme_EnKF2_table0.pdf]

1	newly 3 weeks spec	79.	18.03	{0., 43.}	89.6076	1.50995	newly 3 weeks cumul	342.	142.71	{15., 336.}	84.0088	0.813906	newly 5 weeks spec	40.	5.72	{0., 12.}	92.7	1.85818	newly 5 weeks cumul	434.	157.75	{17., 362.}	82.7535	0.8673	newly 60 weeks cumul	525.	171.11	{17., 459.}	81.781	0.918957
2	newly 3 weeks spec	61.	16.98	{0., 51.}	87.2131	1.52737	newly 3 weeks cumul	211.	89.88	{6., 223.}	88.2275	0.904683	newly 5 weeks spec	24.	7.21	{0., 12.}	98.875	1.65342	newly 5 weeks cumul	275.	108.23	{8., 346.}	87.2473	0.964891	newly 56 weeks cumul	368.	122.93	{8., 435.}	85.3071	1.04601
3	newly 3 weeks spec	93.	10.7	{0., 16.}	93.5269	1.71044	newly 3 weeks cumul	270.	99.49	{10., 129.}	88.6185	0.849183	newly 5 weeks spec	51.	3.33	{0., 7.}	95.6275	2.11045	newly 5 weeks cumul	381.	108.66	{10., 135.}	88.2257	0.951212	newly 58 weeks cumul	577.	114.04	{10., 159.}	87.8856	1.094
4	newly 3 weeks spec	61.	11.94	{0., 22.}	92.0656	1.6844	newly 3 weeks cumul	180.	82.15	{6., 166.}	90.7389	0.857491	newly 5 weeks spec	40.	7.56	{0., 17.}	95.5	1.85146	newly 5 weeks cumul	278.	99.73	{6., 166.}	88.9388	0.969055	newly 57 weeks cumul	490.	122.54	{6., 245.}	87.3959	1.12793
5	newly 3 weeks spec	56.	3.31	{0., 5.}	97.4821	2.33639	newly 3 weeks cumul	216.	48.62	{3., 62.}	93.0093	1.13655	newly 5 weeks spec	40.	0.58	{0., 1.}	98.55	2.40274	newly 5 weeks cumul	294.	50.8	{3., 62.}	92.8095	1.24579	newly 50 weeks cumul	415.	52.31	{3., 62.}	92.6289	1.37134
6	newly 3 weeks spec	54.	13.34	{0., 41.}	91.2222	1.49226	newly 3 weeks cumul	153.	103.97	{12., 312.}	94.6601	0.683251	newly 5 weeks spec	31.	4.8	{0., 9.}	92.129	1.87512	newly 5 weeks cumul	228.	116.6	{12., 401.}	88.807	0.783589	newly 55 weeks cumul	355.	124.38	{12., 402.}	83.0761	0.910047
7	newly 3 weeks spec	68.	19.73	{0., 56.}	92.4853	1.41171	newly 3 weeks cumul	227.	129.21	{10., 272.}	96.7974	0.827691	newly 5 weeks spec	52.	8.37	{0., 16.}	94.0962	1.89149	newly 5 weeks cumul	341.	150.14	{11., 360.}	90.6452	0.922905	newly 59 weeks cumul	479.	170.55	{11., 746.}	88.5073	1.01808
8	newly 3 weeks spec	44.	16.92	{1., 33.}	85.8636	0.85464	newly 3 weeks cumul	184.	133.22	{28., 286.}	85.9022	0.510226	newly 5 weeks spec	25.	4.15	{0., 13.}	87.56	1.4897	newly 5 weeks cumul	249.	144.93	{31., 311.}	83.4659	0.581512	newly 53 weeks cumul	332.	150.4	{34., 388.}	81.3976	0.663826
9	newly 3 weeks spec	65.	17.22	{0., 33.}	94.6769	1.51454	newly 3 weeks cumul	257.	97.44	{11., 218.}	82.716	0.852645	newly 5 weeks spec	29.	6.47	{0., 11.}	96.4483	1.78609	newly 5 weeks cumul	344.	113.82	{12., 247.}	84.6105	0.938086	newly 60 weeks cumul	427.	124.01	{12., 294.}	84.8689	1.00624
10	newly 3 weeks spec	63.	5.13	{0., 8.}	93.4127	1.95164	newly 3 weeks cumul	193.	82.48	{4., 79.}	97.399	0.917033	newly 5 weeks spec	45.	0.73	{0., 2.}	98.3778	2.39235	newly 5 weeks cumul	286.	84.99	{4., 93.}	94.2343	1.05201	newly 53 weeks cumul	451.	85.54	{4., 93.}	92.4967	1.22549
11	newly 3 weeks spec	56.	18.56	{0., 51.}	89.8214	1.30958	newly 3 weeks cumul	197.	139.15	{9., 237.}	107.081	0.824817	newly 5 weeks spec	31.	9.65	{0., 25.}	97.9677	1.59191	newly 5 weeks cumul	268.	160.66	{9., 366.}	100.067	0.887123	newly 59 weeks cumul	345.	188.13	{9., 728.}	97.0754	0.943416
12	newly 3 weeks spec	60.	22.85	{0., 72.}	90.35	1.33371	newly 3 weeks cumul	251.	130.68	{17., 335.}	80.4064	0.697299	newly 5 weeks spec	36.	11.29	{0., 43.}	96.1944	1.71036	newly 5 weeks cumul	329.	157.92	{21., 456.}	80.8207	0.76258	newly 60 weeks cumul	420.	177.91	{23., 528.}	78.4071	0.819648
13	newly 3 weeks spec	43.	14.05	{0., 33.}	99.6977	1.53869	newly 3 weeks cumul	128.	120.21	{11., 266.}	116.789	0.644094	newly 5 weeks spec	32.	4.79	{0., 9.}	96.4688	2.0199	newly 5 weeks cumul	184.	132.83	{11., 376.}	106.929	0.740494	newly 53 weeks cumul	478.	142.99	{12., 470.}	88.4038	1.03613
14	newly 3 weeks spec	35.	10.35	{0., 16.}	90.6571	1.18408	newly 3 weeks cumul	175.	74.57	{10., 151.}	84.2	0.76167	newly 5 weeks spec	20.	2.85	{0., 7.}	93.45	1.64757	newly 5 weeks cumul	217.	82.88	{10., 154.}	84.3502	0.808295	newly 57 weeks cumul	250.	88.75	{10., 154.}	84.332	0.841169
15	newly 3 weeks spec	59.	12.37	{0., 15.}	97.0339	1.64648	newly 3 weeks cumul	186.	90.88	{9., 86.}	97.0753	0.839184	newly 5 weeks spec	32.	2.59	{0., 7.}	94.0938	2.06052	newly 5 weeks cumul	268.	99.08	{9., 102.}	94.6418	0.954371	newly 52 weeks cumul	342.	102.75	{9., 154.}	92.8275	1.03518
16	newly 3 weeks spec	56.	21.74	{0., 87.}	98.9286	1.42367	newly 3 weeks cumul	175.	145.53	{7., 608.}	118.394	0.820619	newly 5 weeks spec	29.	6.49	{0., 13.}	93.1379	1.70645	newly 5 weeks cumul	242.	164.37	{7., 754.}	109.293	0.893205	newly 59 weeks cumul	393.	173.77	{7., 772.}	95.4071	1.0291
17	newly 3 weeks spec	48.	15.42	{0., 45.}	86.625	1.21421	newly 3 weeks cumul	177.	110.08	{12., 255.}	98.3277	0.752066	newly 5 weeks spec	42.	6.67	{0., 19.}	92.0714	1.82657	newly 5 weeks cumul	264.	126.16	{14., 386.}	92.4015	0.849926	newly 56 weeks cumul	488.	143.01	{15., 408.}	86.252	1.02952
18	newly 3 weeks spec	77.	19.91	{0., 51.}	91.9091	1.45451	newly 3 weeks cumul	277.	134.28	{12., 272.}	96.5343	0.874353	newly 5 weeks spec	51.	7.8	{0., 16.}	94.0392	1.90515	newly 5 weeks cumul	383.	154.55	{13., 481.}	92.6397	0.950901	newly 59 weeks cumul	532.	170.34	{13., 779.}	89.4248	1.04467
19	newly 3 weeks spec	85.	20.83	{0., 61.}	91.3529	1.47694	newly 3 weeks cumul	252.	124.51	{14., 376.}	85.6865	0.745464	newly 5 weeks spec	73.	9.92	{0., 17.}	92.9315	1.94222	newly 5 weeks cumul	405.	148.74	{14., 532.}	83.7975	0.872304	newly 60 weeks cumul	563.	165.38	{14., 684.}	82.7211	0.96701
20	newly 3 weeks spec	46.	11.4	{0., 19.}	91.6957	1.33843	newly 3 weeks cumul	195.	82.16	{7., 127.}	86.6462	0.765739	newly 5 weeks spec	24.	2.83	{0., 5.}	92.7083	1.7157	newly 5 weeks cumul	257.	91.12	{10., 164.}	86.3969	0.832189	newly 53 weeks cumul	327.	94.13	{10., 168.}	86.7431	0.914439
21	newly 3 weeks spec	59.	12.74	{0., 11.}	94.5763	1.51678	newly 3 weeks cumul	155.	99.18	{5., 330.}	109.961	0.896285	newly 5 weeks spec	46.	4.79	{0., 7.}	96.8478	2.07607	newly 5 weeks cumul	257.	112.03	{6., 513.}	98.1907	1.02427	newly 56 weeks cumul	478.	121.1	{6., 580.}	88.9247	1.20789
22	newly 3 weeks spec	49.	6.71	{0., 16.}	89.7347	1.58225	newly 3 weeks cumul	172.	94.1	{6., 141.}	99.2907	0.829097	newly 5 weeks spec	32.	3.64	{0., 8.}	94.0625	1.92023	newly 5 weeks cumul	242.	102.38	{6., 150.}	93.5124	0.908744	newly 57 weeks cumul	358.	119.61	{6., 209.}	90.3883	1.00851
23	newly 3 weeks spec	51.	18.25	{0., 46.}	97.8235	1.32139	newly 3 weeks cumul	194.	128.5	{11., 244.}	104.062	0.807162	newly 5 weeks spec	37.	7.95	{0., 10.}	99.3784	1.817	newly 5 weeks cumul	294.	148.38	{11., 416.}	97.3946	0.917737	newly 59 weeks cumul	412.	168.02	{12., 782.}	94.2864	1.01344
24	newly 3 weeks spec	65.	15.32	{0., 40.}	89.8154	1.47964	newly 3 weeks cumul	306.	161.74	{26., 475.}	88.5425	0.737796	newly 5 weeks spec	35.	5.08	{0., 8.}	94.9714	1.81501	newly 5 weeks cumul	386.	174.01	{28., 547.}	84.4948	0.781486	newly 60 weeks cumul	448.	186.78	{28., 818.}	83.1429	0.821266
25	newly 3 weeks spec	55.	9.91	{0., 15.}	89.4	1.19514	newly 3 weeks cumul	189.	56.92	{8., 85.}	83.2487	0.791943	newly 5 weeks spec	45.	5.05	{0., 7.}	95.1778	1.70558	newly 5 weeks cumul	280.	69.08	{9., 103.}	86.5357	0.89564	newly 57 weeks cumul	488.	80.42	{10., 120.}	88.8893	1.07704
26	newly 3 weeks spec	68.	18.12	{0., 69.}	91.0882	1.44786	newly 3 weeks cumul	336.	155.64	{8., 597.}	97.3333	0.985027	newly 5 weeks spec	22.	4.3	{0., 7.}	93.9091	1.63011	newly 5 weeks cumul	403.	167.9	{9., 717.}	93.8859	1.01214	newly 59 weeks cumul	451.	175.24	{9., 717.}	91.0687	1.02805
27	newly 3 weeks spec	39.	14.11	{0., 23.}	97.2564	1.26637	newly 3 weeks cumul	136.	87.7	{7., 115.}	101.588	0.716506	newly 5 weeks spec	42.	5.89	{0., 11.}	89.4048	1.75235	newly 5 weeks cumul	218.	102.77	{7., 168.}	94.9128	0.83079	newly 54 weeks cumul	436.	119.84	{7., 244.}	89.4083	1.04809
28	newly 3 weeks spec	51.	13.16	{0., 21.}	93.9216	1.4551	newly 3 weeks cumul	228.	107.57	{11., 167.}	90.9254	0.771429	newly 5 weeks spec	34.	6.01	{0., 12.}	92.5588	1.59165	newly 5 weeks cumul	314.	122.48	{11., 209.}	88.586	0.828325	newly 58 weeks cumul	442.	135.22	{11., 245.}	85.9457	0.902085
29	newly 3 weeks spec	80.	17.6	{0., 30.}	95.25	1.77202	newly 3 weeks cumul	289.	159.98	{7., 614.}	90.4844	0.881173	newly 5 weeks spec	36.	6.99	{0., 11.}	95.5278	1.81962	newly 5 weeks cumul	356.	178.09	{7., 676.}	87.0534	0.90839	newly 55 weeks cumul	420.	191.93	{7., 710.}	84.0738	0.936526
30	newly 3 weeks spec	25.	14.12	{0., 41.}	88.16	1.04608	newly 3 weeks cumul	94.	145.2	{12., 228.}	134.128	0.440969	newly 5 weeks spec	18.	6.74	{0., 11.}	101.111	1.4149	newly 5 weeks cumul	134.	161.73	{12., 286.}	117.201	0.512778	newly 50 weeks cumul	194.	177.7	{12., 363.}	106.268	0.61118
31	newly 3 weeks spec	69.	21.1	{0., 59.}	92.6377	1.43822	newly 3 weeks cumul	249.	146.6	{6., 414.}	99.1647	0.891488	newly 5 weeks spec	42.	8.99	{0., 20.}	94.881	1.75577	newly 5 weeks cumul	339.	169.39	{6., 506.}	93.9263	0.955025	newly 59 weeks cumul	419.	188.9	{6., 779.}	91.9045	1.01136
32	newly 3 weeks spec	64.	22.07	{0., 77.}	90.3594	1.49623	newly 3 weeks cumul	288.	145.31	{6., 499.}	90.3854	0.93551	newly 5 weeks spec	34.	10.27	{0., 32.}	94.6176	1.72047	newly 5 weeks cumul	366.	169.55	{6., 614.}	88.041	0.980803	newly 59 weeks cumul	414.	198.04	{6., 816.}	89.6087	1.00932
33	newly 3 weeks spec	77.	9.61	{0., 16.}	92.0649	1.92942	newly 3 weeks cumul	224.	123.38	{10., 464.}	91.7232	0.778569	newly 5 weeks spec	47.	3.05	{0., 6.}	94.0213	1.98037	newly 5 weeks cumul	333.	131.55	{11., 469.}	83.7387	0.860058	newly 55 weeks cumul	564.	140.64	{12., 485.}	80.3511	1.012
34	newly 3 weeks spec	60.	9.7	{0., 18.}	93.0333	1.68775	newly 3 weeks cumul	219.	100.08	{13., 121.}	92.8767	0.793421	newly 5 weeks spec	38.	4.69	{0., 8.}	94.1842	1.80992	newly 5 weeks cumul	306.	111.44	{14., 149.}	90.7712	0.870188	newly 58 weeks cumul	433.	124.23	{15., 163.}	89.1709	0.960641
35	newly 3 weeks spec	68.	17.44	{0., 48.}	92.2941	1.47093	newly 3 weeks cumul	222.	138.34	{6., 244.}	106.694	0.912939	newly 5 weeks spec	42.	8.86	{0., 9.}	98.1429	1.83152	newly 5 weeks cumul	317.	159.11	{8., 489.}	99.858	1.00076	newly 59 weeks cumul	467.	179.18	{8., 793.}	94.3255	1.12125
36	newly 3 weeks spec	69.	12.67	{0., 29.}	85.9565	1.3648	newly 3 weeks cumul	224.	83.28	{16., 138.}	85.1161	0.785887	newly 5 weeks spec	46.	5.13	{0., 14.}	91.8913	1.82866	newly 5 weeks cumul	333.	96.52	{16., 189.}	84.2523	0.885355	newly 56 weeks cumul	462.	111.62	{17., 259.}	83.8139	0.972028
37	newly 3 weeks spec	58.	5.59	{0., 11.}	92.431	1.79152	newly 3 weeks cumul	192.	65.44	{4., 139.}	89.	0.969957	newly 5 weeks spec	31.	1.53	{0., 4.}	95.2581	2.12761	newly 5 weeks cumul	273.	70.05	{4., 140.}	87.3297	1.07272	newly 51 weeks cumul	413.	76.57	{4., 182.}	87.615	1.2092
38	newly 3 weeks spec	60.	13.74	{0., 35.}	84.6667	1.34359	newly 3 weeks cumul	244.	81.88	{4., 225.}	81.4016	0.9067	newly 5 weeks spec	40.	5.44	{0., 11.}	94.5	1.78095	newly 5 weeks cumul	319.	96.25	{6., 256.}	82.21	0.962718	newly 56 weeks cumul	406.	110.9	{6., 287.}	83.5813	1.02001
39	newly 3 weeks spec	93.	21.5	{0., 54.}	92.5161	1.52769	newly 3 weeks cumul	314.	130.17	{7., 290.}	93.9841	0.980062	newly 5 weeks spec	46.	8.22	{0., 19.}	93.8261	1.89644	newly 5 weeks cumul	440.	151.9	{8., 529.}	90.1955	1.06346	newly 59 weeks cumul	558.	169.85	{8., 717.}	87.9624	1.12623
40	newly 3 weeks spec	44.	10.32	{0., 18.}	97.0909	1.68734	newly 3 weeks cumul	165.	91.95	{8., 128.}	96.8788	0.753086	newly 5 weeks spec	31.	4.63	{0., 6.}	94.8065	1.92423	newly 5 weeks cumul	230.	103.46	{12., 155.}	94.0957	0.839071	newly 57 weeks cumul	342.	118.8	{12., 233.}	91.0643	0.95864
41	newly 3 weeks spec	54.	10.23	{0., 17.}	93.0556	1.59154	newly 3 weeks cumul	164.	73.84	{8., 102.}	91.7073	0.80124	newly 5 weeks spec	33.	3.35	{0., 4.}	95.7273	1.97093	newly 5 weeks cumul	248.	83.08	{8., 106.}	89.5484	0.924842	newly 52 weeks cumul	369.	88.16	{8., 113.}	88.2439	1.05939
42	newly 3 weeks spec	75.	29.09	{0., 139.}	95.7467	1.54822	newly 3 weeks cumul	284.	142.56	{16., 493.}	82.4718	0.722725	newly 5 weeks spec	55.	12.97	{0., 44.}	91.6182	1.82567	newly 5 weeks cumul	401.	175.87	{16., 624.}	82.6708	0.815531	newly 60 weeks cumul	505.	199.74	{16., 768.}	82.3208	0.879554
43	newly 3 weeks spec	82.	19.9	{0., 74.}	88.2439	1.58623	newly 3 weeks cumul	252.	159.	{7., 788.}	108.048	0.928948	newly 5 weeks spec	62.	7.9	{0., 12.}	93.4839	2.0358	newly 5 weeks cumul	378.	178.64	{7., 788.}	95.7566	1.01483	newly 59 weeks cumul	545.	197.98	{8., 802.}	88.9321	1.10853
44	newly 3 weeks spec	70.	11.07	{0., 15.}	94.7857	1.68759	newly 3 weeks cumul	222.	76.64	{5., 118.}	89.8559	0.947024	newly 5 weeks spec	43.	4.5	{0., 11.}	93.2558	1.81188	newly 5 weeks cumul	326.	87.57	{5., 153.}	89.5613	1.04127	newly 57 weeks cumul	430.	96.95	{5., 169.}	88.3651	1.09651
45	newly 3 weeks spec	80.	18.08	{0., 52.}	87.825	1.49731	newly 3 weeks cumul	274.	121.19	{23., 282.}	81.0328	0.733967	newly 5 weeks spec	45.	7.82	{0., 20.}	92.1333	1.8185	newly 5 weeks cumul	386.	141.26	{23., 378.}	79.9637	0.803192	newly 60 weeks cumul	569.	156.13	{24., 397.}	81.7557	0.931746
46	newly 3 weeks spec	54.	9.28	{0., 17.}	92.1111	1.63789	newly 3 weeks cumul	225.	78.13	{8., 104.}	89.4444	0.89572	newly 5 weeks spec	46.	5.65	{0., 14.}	95.6739	1.92819	newly 5 weeks cumul	335.	90.36	{10., 135.}	88.0299	0.996456	newly 57 weeks cumul	485.	104.48	{10., 218.}	87.1753	1.08849
47	newly 3 weeks spec	69.	10.26	{0., 18.}	93.971	1.71694	newly 3 weeks cumul	202.	102.15	{11., 140.}	91.5594	0.767755	newly 5 weeks spec	59.	3.12	{0., 5.}	96.3051	2.23619	newly 5 weeks cumul	320.	111.02	{11., 149.}	89.1438	0.908984	newly 58 weeks cumul	544.	116.92	{11., 159.}	88.0993	1.09584
48	newly 3 weeks spec	53.	13.28	{0., 20.}	95.1321	1.55153	newly 3 weeks cumul	180.	101.28	{7., 174.}	96.6222	0.798153	newly 5 weeks spec	40.	5.65	{0., 10.}	93.675	1.80436	newly 5 weeks cumul	270.	115.59	{8., 223.}	92.5593	0.885092	newly 58 weeks cumul	460.	129.03	{9., 260.}	87.3761	1.00516
49	newly 3 weeks spec	56.	26.23	{0., 102.}	94.4821	1.32642	newly 3 weeks cumul	258.	152.59	{21., 477.}	82.9264	0.691603	newly 5 weeks spec	43.	11.57	{0., 38.}	92.7209	1.78142	newly 5 weeks cumul	341.	182.74	{21., 675.}	82.9384	0.756209	newly 60 weeks cumul	475.	202.64	{22., 747.}	80.1179	0.843815
50	newly 3 weeks spec	49.	8.77	{0., 16.}	87.0816	1.41816	newly 3 weeks cumul	150.	77.31	{8., 132.}	85.6067	0.700331	newly 5 weeks spec	26.	4.91	{0., 6.}	97.8077	1.69644	newly 5 weeks cumul	211.	88.41	{8., 234.}	85.0095	0.784753	newly 58 weeks cumul	361.	100.98	{11., 271.}	84.0222	0.933273
